# Mutation of *OsSAC3*, Encoding the Xanthine Dehydrogenase, Caused Early Senescence in Rice

**DOI:** 10.3390/ijms231911053

**Published:** 2022-09-21

**Authors:** Ziyu Xie, Bingbing Zhao, Mengxue Zhang, Xianchun Sang, Fangming Zhao, Ping Feng, Guanghua He, Xiaoyan Zhu

**Affiliations:** Rice Research Institute, Key Laboratory of Application and Safety Control of Genetically Modified Crops, Southwest University, Chongqing 400715, China

**Keywords:** *OsSAC3*, leaf senescence, sugar accumulation, xanthine dehydrogenase, uric acid, *Oryza* *sativa*

## Abstract

In both animals and higher plants, xanthine dehydrogenase is a highly conserved housekeeping enzyme in purine degradation where it oxidizes hypoxanthine to xanthine and xanthine to uric acid. Previous reports demonstrated that xanthine dehydrogenase played a vital role in N metabolism and stress response. Is xanthine dehydrogenase involved in regulating leaf senescence? A recessive early senescence mutant with excess sugar accumulation, *ossac3*, was isolated previously by screening the EMS-induced mutant library. Here, we show that xanthine dehydrogenase not only plays a role in N metabolism but also involved in regulating carbon metabolism in rice. Based on map-based cloning, OsSAC3 was identified, which encodes the xanthine dehydrogenase. *OsSAC3* was constitutively expressed in all examined tissues and the OsSAC3 protein located in the cytoplasm. Transcriptional analysis revealed purine metabolism, chlorophyll metabolism, photosynthesis, sugar metabolism and redox balance were affected in the *ossac3* mutant. Moreover, carbohydrate distribution was changed, leading to the accumulation of sucrose and starch in the leaves containing *ossac3* on account of decreased expression of *OsSWEET3a*, *OsSWEET6a* and *OsSWEET14* and oxidized inactivation of starch degradation enzymes in *ossac3*. These results indicated that *OsSAC3* played a vital role in leaf senescence by regulating carbon metabolism in rice.

## 1. Introduction 

Leaf senescence is a complex physiological process, which is not only influenced by external environment factors such as temperature, light, drought, nutrient deficiency, wounding and pathogen infection [1], but also affected by internal genetic factors such as the developmental stage and phytohormone levels [1]. Premature leaf senescence has a direct impact on crop yields by changing the duration of photosynthesis and modifying the nutrient remobilization efficiency and harvest index. Therefore, understanding the molecular mechanism of leaf senescence is important for breeders to raise crop production and quality. Many leaf-senescence-associated genes have been identified in rice. These genes can be divided into different categories according to the metabolic pathways. NYC1, NYC3, NYC4, OsPAO, OsRCCR1 and OsSGR participate in chloroplast development and chlorophyll metabolism [1]. OsCOI1b, OsFBK12 and OsPLS1 participate in plant hormone signaling [1]. OsGATA12, OsWRKY42, OsHox33, and OsNAC106 are transcription factors [1]. OsSRT1, OsSRT2 and LTS1 participate in energy metabolism pathways [1]. Carbon metabolism also participates in regulating leaf senescence [2,3,4]. Plant hexokinase functions as a catalyst which phosphorylates Glc, and as a glucose sensor. A mutant in *hexokinase* 1 (*HXK1*), the *gin2-1* mutant, shows delayed senescence [3], and over-expression of the *Arabidopsis HXK1* in tomato accelerates senescence [2]. Moreover, strong accumulation of Trehalose 6-phosphate (T6P) was found in senescencing *Arabidopsis* leaves, in parallel with a rise in sugar contents [4]. In addition, the *OsSAC1* gene encodes an endoplasmic reticulum protein with unknow function, which also participates in regulating leaf senescence; the *ossac1* mutant showed early senescence with yellowish leaves on account of starch accumulation in the chloroplast [5]. Leaf senescence of rice is a complex process which involves many genes and metabolic pathways. Although great progress has been made on rice leaf senescence research, the molecular mechanisms of leaf senescence still remain unclear and further studies on this subject should be carried out.

Xanthine dehydrogenase (XDH) is a ubiquitous molybdenum-iron-flavo enzyme with a central role in purine catabolism, where it catalyzes the oxidation of hypoxanthine to xanthine and from xanthine to uric acid. The fully constituted enzyme from eukaryotes is a homodimer composed of two identical subunits of about 145 kDa, each being subdivided into three domains: an N-terminal domain of 20 kDa for binding of two non-identical iron-sulfur clusters of the [2Fe-2S] type, a 40 kDa domain harboring a FAD-binding site, and a C-terminal aldehyde oxidase domain [6]. Accounting to its central role in purine metabolism, a deficiency in *XDH* genes leads to serious growth defects in various species. The *rosy* mutant of *Drosophila melanogaster* was unable to synthesize urate because of no detectable of XDH activity [7]. A point mutation in the structural gene for xanthine dehydrogenase of *Aspergillus nidulans* results in several dramatic pleiotropic effects, including: being completely unable to utilize hypoxanthine as their nitrogen source, and resistance to the irreversible inhibitor allopurinol [8]. In humans, the enzyme is the target of therapeutic drugs against hyperuricemia or gout. Mutation of XDH gene results in classical xanthinuria in human [9]. Two highly homologous genes, *AtXDH1* and *AtXDH2*, encode the XDH protein in Arabidopsis. Total XDH protein levels were completely reduced when both *AtXDH1* and *AtXDH2* were both silenced, which resulted in the dramatic overaccumulation of xanthine and a reduced growth phenotype [10]. The above results revealed that XDH mainly functions in N metabolism and various stress responses. However, XDH’s function in carbon metabolism has not been reported.

A recessive mutant exhibited an early senescent phenotype, *ossac3*, which was identified previously [11]. Stroma lamella degradation caused by excess starch accumulation in the chloroplast might be the main reason leading to the early senescence in *ossac3*. In this study, the *OsSAC3* was map-based, cloned and identified, which encoded the classic xanthine dehydrogenase. *OsSAC3* was constitutively expressed in all examined tissues. Moreover, the OsSAC3 protein was localized in the cytoplasm. Furthermore, transcriptomic analysis revealed that not only purine metabolism but also carbon metabolism was affected in *ossac3*. These results indicated that *OsSAC3* plays a vital role in regulating carbon metabolism.

## 2. Results

### 2.1. Map-Based Clone and Identification of OsSAC3

**Figure 1:**
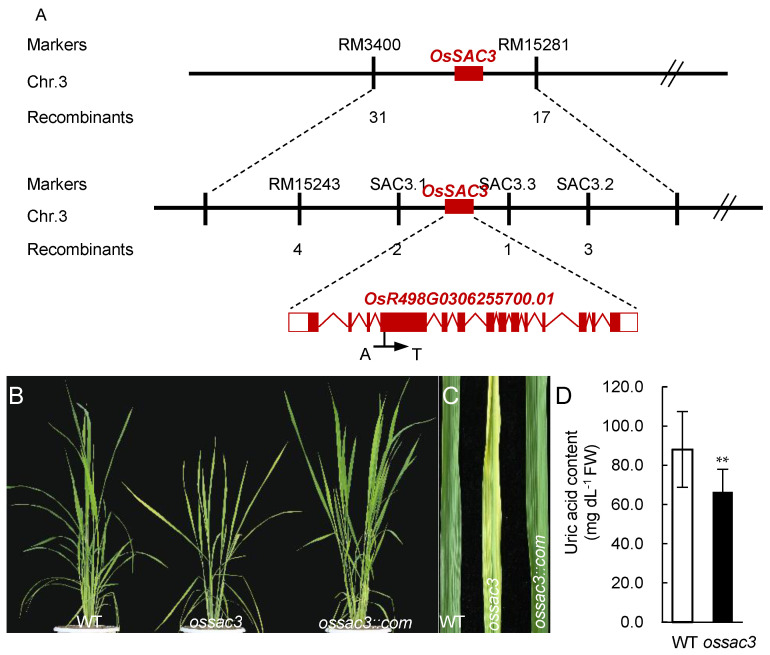
Map-based clone and identification of *OsSAC3*. (**A**): Map-based clone of *OsSAC3*; (**B**,**C**): Complementation of *ossac3* mutants with the wild-type *OsSAC3* genomic fragments; (**D**): Measurement of uric acid content between the wild type and the *ossac3* mutants. Decreased uric acid was observed in the *ossac3′s* leaves than that of the wild type. ** *p* < 0.001 determined by Student’s *t*-test.

The *ossac3* mutant, which displayed early senescence with sugar (starch and sucrose) accumulation from the seedling stage, has been previously isolated and characterized [11]. The *OsSAC3* was mapped to a 374.2 kb region between the simple sequence repeat (SSR) marker RM3400 and RM15281 on chromosome 3 [11]. To further fine-map and clone the *OsSAC3* gene, 5 new polymorphic SSR marker and another 450 recessive F_2_ plants were obtained and applied. Ultimately, the *OsSAC3* gene was mapped in a 252.7 kb region between SSR marker SAC3.1 and SAC3.3 (Figure 1A). According to http://www.mbkbase.org/rice (accessed on 1 January 2018), 10 genes were predicted in this mapping region, and sequence analysis revealed a single nucleotide substitution from A to T was observed in the fourth exon on *OsR498G0306255700.01* in *ossac3* (Figure 1A), which resulted in an amino acid substitution form His124 to Leu (Figure 1A). To verify that this mutation caused the early senescence with excess sugar accumulation mutational phenotype, complementary transgenic plants of *ossac3* (*OsSAC3*::COM) were obtained by expressing the wild type (WT) *OsSAC3* gene under the control of the *Cauliflower mosaic virus 35S* (*CaMV35S*) promoter in the *ossac3* background. The heterozygous *OsSAC3*::COM plants resembled the normal phenotypes of the WT plants (Figure 1B,C). These results indicated that *OsR498G0306255700.01* was the *OsSAC3* gene.

### 2.2. Bioinformatic Analysis of OsSAC3

**Figure 2:**
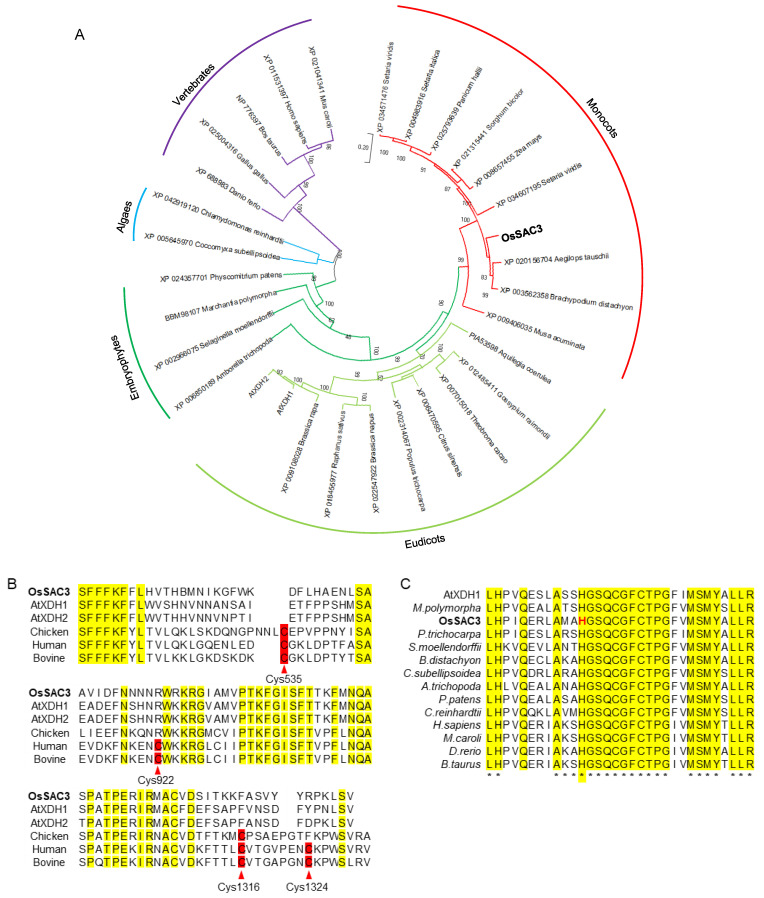
Bioinformatic analysis of OsSAC3. (**A**): Phylogenetic analysis of OsSAC3 revealed that the xanthine dehydrogenase, encoded by OsSAC3 was conserved among the eukaryotes and was classified into the monocots; (**B**): The four Cys residues (Cys 535, 922 1316 1324) functioned in the transition from xanthine dehydrogenase to xanthine oxidase were missing in OsSAC3, AtXDH1/2 and Chicken (XP_025004316); (**C**): Conserved sequence of [2Fe-2S] center in OsSAC3 and H124, marked in red color, was mutated into Leu in *ossac30*; *M. polymorpha* (*Marchantia polymorpha*, BBM98107), *P. trichocarpa* (*Populus trichocarpa*, XP_002314067), *S. moellendorffii* (*Selaginella moellendorffii*, XP_002966075), *B. distachyon* (XP_003562358, *Brachypodium distachyon*), *C. illinoinensis* (*Carya illinoinensis*, XP_042959708), *A. trichopoda* (*Amborella trichopoda*, XP_006850189), *P. patens* (*Physcomitrium patens*, XP_024357701), *C. reinhardtii* (*Chlamydomonas reinhardtii*, XP_042919120), *H. sapiens* (*Homo sapiens*, XP_011531397), *M. caroli* (*Mus caroli*, XP_021041341), *D. rerio* (*Danio rerio*, XP_688983), *B. taurus* (*Bos taurus*, NP_7*76397*); * represents highly conserved.

Bioinformatic analysis indicated that the *OsSAC3* gene contained 14 exons and 13 introns, encoding the classic xanthine dehydrogenase with 150.2 kDa and a pI of 6.99 and containing three conserved domains: the non-identical iron-sulfur clusters of the [2Fe-2S] type, the FAD-binding domain and the C-terminal aldehyde oxidase domain. The results of a BLAST search indicated that there could be a single copy of the *OsSAC3* gene in the rice genome because *OsSAC3* shared only 43% amino acid identity with its closest homolog *OsR498G1018112500.01*. Furthermore, OsSAC3 was conserved in eukaryotes (Figure 2A). Mammalian XDH exists in two interconvertible forms: the xanthine dehydrogenase form with high reactivity toward NAD^+^ as the electron acceptor, and the xanthine oxidase form with high reactivity toward O_2_ as the electron acceptor [12,13]. Four cysteines (cys535, 992, 1316, 1324) contributed to the conformational change from the xanthine dehydrogenase form to the xanthine oxidase form in rats; they were conserved in humans and bovine, but were all missing in chicken, *Arabidopsis* and rice (Figure 2B) [12,13].

Furthermore, His124 was conserved in the [2Fe-2S] iron-sulfur clusters which mediated electron transferring from molybdenum to FAD and located near the fifth conserved cysteine in the [2Fe-2S] center (Figure 2B) [14]. The electron transferring function might be damaged, accounting to the change from His124 to Leu in *ossac3* (Figure 2C). This resulted in defects of xanthine dehydrogenase activity. To confirm this speculation and to verify *OsSAC3′s* xanthine dehydrogenase activity, uric acid content was analyzed in both the WT and the *ossac3* mutant. Reduced uric acid content was observed in the *ossac3* compared with the WT (Figure 1D) indicated that *OsSAC3* functioned the xanthine dehydrogenase activity in rice, and that His124 is essential for its activity.

### 2.3. OsSAC3 Encodes a Cytoplasmic Xanthine Dehydrogenase with Constitutively Expression Pattern

**Figure 3:**
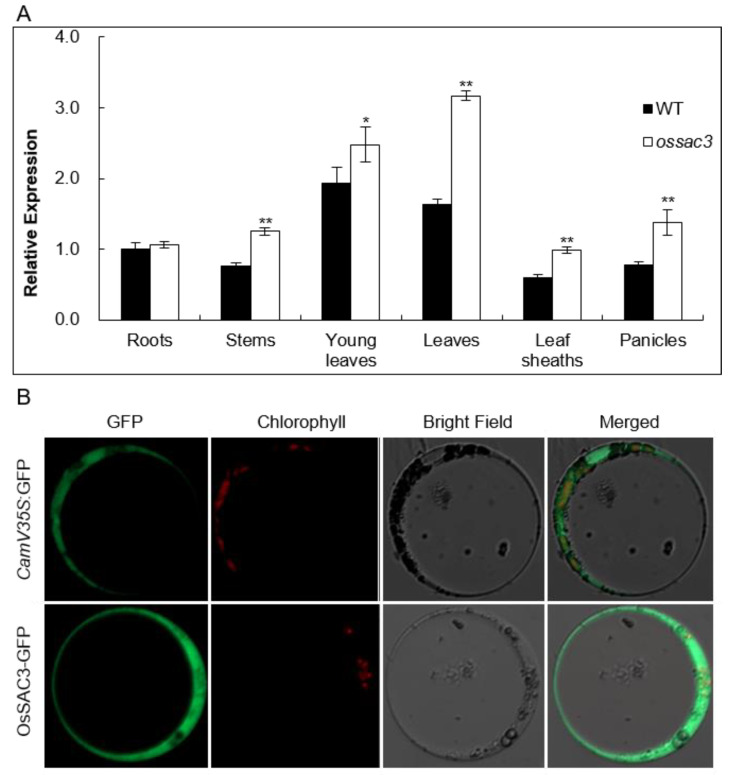
Expression and subcellular analysis of OsSAC3. (**A**): Expression pattern of *OsSAC3*; *OsSAC3* was expressed in roots, stems, leaves, leaf sheathes and panicles. Additionally, increased expression of *OsSAC3* was observed in the *ossac3* than the wild type (WT). Three biological repeats were conducted. * and ** represent *p* < 0.05 and *p* < 0.001, respectively, determined by Student’s *t*-test; (**B**): Subcellular analysis of OsSAC3. OsSAC3 was located in the cytoplasm.

Quantitative real-time PCR (qRT-PCR) was used to examine *OsSAC3* expression in various WT and *ossac3* tissues, including the roots, stems, young leaves, old leaves, leaf sheathes and panicles. *OsSAC3* transcripts were observed in all tissues examined in both the WT and *ossac3*, and the expression level of *OsSAC3* in *ossac3* was higher than those of the WT (Figure 3A). These results revealed that *OsSAC3* was constitutively expressed in all tissues in rice.

An OsSAC3-GFP fusion plasmid under the control of the *CaMV35S* promoter was constructed to investigate the subcellular location of OsSAC3. The fusion construct was transferred into rice protoplasts by polyethylene glycol (PEG)-mediated transformation. Additionally, the fluorescent signal was observed in the cytoplasm (Figure 3B). The above results indicated that OsSAC3 was the xanthine dehydrogenase in the cytoplasm.

### 2.4. Transcriptome and Gene Ontology Enrichment Analysis

**Figure 4:**
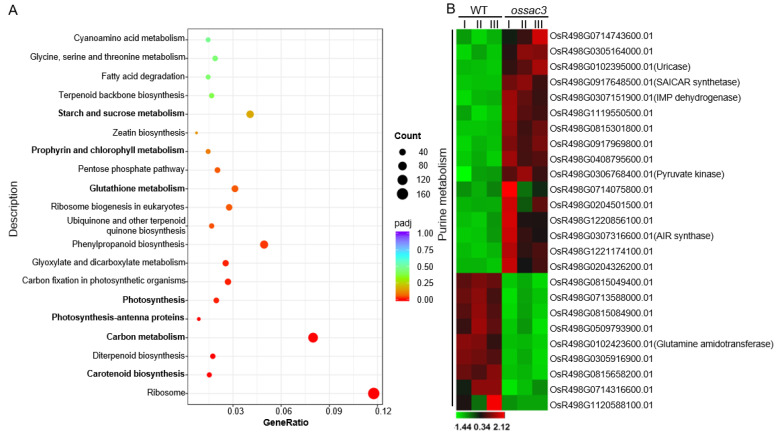
Transcriptome analysis between the wild type (WT) and the *OsSAC3* mutant. (**A**): KEGG enrichment of differentially expressed genes in *OsSAC3* transcriptome data. The enriched pathways are listed on the left; (**B**): Heatmap showing the expression patterns of selected genes in the process of purine metabolism related process. Data are from three biological replicates (I, II and III).

To further explore the function of *OsSAC3*, the transcriptome sequencing of the expanded early senescent leaves from the *ossac3* and the WT was performed. A significant positive correlation among three biological replicates (Pearson’s correlation > 0.655) was observed. After filtering, a total of 359,073,332 paired-end reads were obtained, and each biological replicate was uniquely mapped to the rice reference genome. The RNA-seq results showed that gene expression was altered significantly in *ossac3* and WT plants. The differentially expressed genes (DEGs) were identified using the screening standards FDR < 0.05 and |log2FC| > 1. A total of 8144 DEGs between the *ossac3* mutant and WT were identified, of which 4413 genes were up-regulated and 3731 were down-regulated (Supplementary Table S1). The results of KEGG analysis showed that genes in ribosome, oxidation-reduction process, sugar metabolic process, photosynthetic pigments metabolism, and photosynthesis were differentially expressed (Figure 4A).

### 2.5. Gene Expression Related to Purine Metabolism Was Altered in ossac3

Overall, 14 enzymes participate in the de novo synthesis of purine nucleotides [15]. PRPP amidotransferase (*OsR498G0102423600.01*) starting the purine biosynthesis was down-regulated in *ossac3* (Figure 4B). N-succinyl-5-aminoimidazole-4-carboxamide ribonucleotide (SAICAR) synthase (*OsR498G0917648500.01*) catalyzing the formation of SAICAR was up-regulated in *ossac3* (Figure 4B). Adenylate kinase catalyzes the reversible transphosphorylation reaction interconverting AMP to ADP and ATP, which is considered a key step in energy metabolism and is essential to recycle AMP, ADP and ATP [15]. In total, 14 genes encoding adenylate kinase were found by searching http://www.mbkbase.org/rice (accessed on 1 March 2021). Among them, *OsR498G0815049400.01* and *OsR498G1221174100.01* were down- and up-regulated, respectively (Figure 4B). *OsR498G0714316600.01* encoding the purine salvage enzyme adenine phosphoribosyltranserase (APTs) was down-regulated in *ossac3* (Figure 4B) [15]. These results indicated that the expression of genes in the purine metabolism was altered in *ossac3*.

### 2.6. Redox Process Was Altered in ossac3

**Figure 5:**
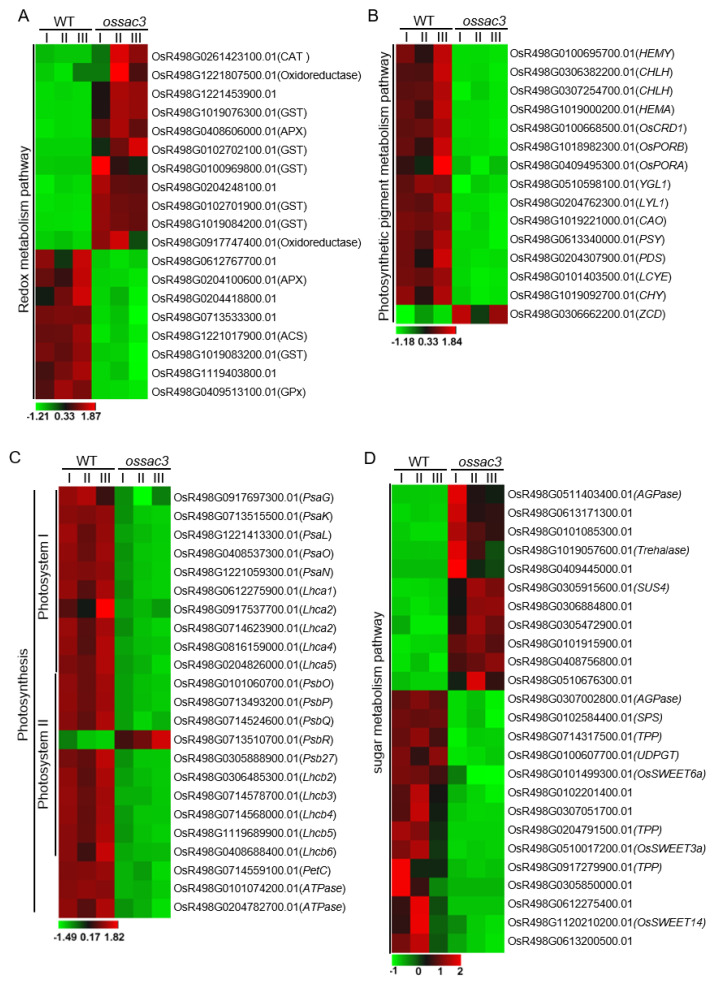
Redox metabolism, photosynsthesis and sugar metabolism were affected in *ossac3*. (**A**–**D**): Heatmap showing the expression patterns of selected genes in the process of redox metabolism (**A**), photosynthetic pigment metabolism (**B**), photosynthesis (**C**) and sugar metabolism (**D**) related process. Data are from three biological replicates (I, II and III).

Reactive oxygen species (ROS) (e.g., O_2_−^.^, −OH, H_2_O_2_ and ^1^O_2_) are mainly generated from the chloroplast, mitochondria and peroxisome, and have numerous toxic and harmful effects due to their high reactivity [16]. Plant cells have developed a complex antioxidant machinery for ROS scavenging which is vital for plant development and growth. In this study, numerous nuclear genes encoding products with ROS scavenging are differentially affected in *ossac3* mutants (Figure 5A).

Plant peroxidases are heme-containing proteins that catalyze the reduction of H_2_O_2_ by taking electrons to various donor molecules [17,18]. The rice genome contains 138 peroxidase genes, and 24 of these genes are differentially regulated in *ossac3* compared with WT. Most of those differentially expressed peroxidase genes showed increased expression levels in *ossac3* (Figure 5A). Catalase is another major ROS-scavenging enzyme in all aerobic organisms [17,18]. The *ossac3* mutant exhibits two-fold changes in the expression of *OsR498G0261423100.01* which encodes OsCATA (Figure 5A).

GSTs are typical Glutathione-dependent enzymes of the antioxidant system [19,20]. At least 79 GST genes were presented in the rice genome and grouped into seven classes by phylogenetic analysis [19,20]. In this study, several GST-encoding genes were differentially regulated in *ossac3* compared with WT, and most of these differentially expressed GSTs genes exhibited increased expression levels (Figure 5A). Both *OsGSTU4* (*OsR498G1019079100.01*) and OsGSTU6 (*OsR498G0101330600.01*) belong to the Tau classes, localized in the cytosol and nucleus of cells, and regulate salinity and oxidative stresses and cadmium (Cd) stress tolerance, respectively [21]. The *ossac3* mutant exhibited a 10- and 3.4-fold change in the expression of *OsGSTU4* and *OsGSTU6*, respectively, when compared with the WT [21,22]. *OsGSTF5* (*OsR498G0100969800.01*) belonged to the Phi classes of GSTs and played a role in the conjugation of herbicide via its glutathione peroxidase activity [23]. The *ossac3* mutant exhibited 5.2-fold changes in the expression of *OsGSTF5* when compared with the WT (Figure 5A). *OsGSTF6* (*OsR498G1019126800.01*) also belonged to the Phi classes of GSTs and was reported to be an active GST restricted to the phloem region of normal rice leaves [22]. Additionally, OsGSTF6 also showed increased expression levels in the *ossac3* (Figure 5A). Although the *ossac3* mutant exhibited decreased POD, SOD and CAT activity in our previous study [11], these results indicated that ROS-scavenger encoding genes were induced when *OsSAC3* mutated.

### 2.7. Photosynthesis Was Damaged in ossac3

Photosynthesis is the basis of all life on earth. Different expression pattern of photosynthesis-related genes was analyzed. Genes involved in chlorophyll biogenesis including *HEMY*, *CHLH*, *HEMA*, *OsCRD1*, *OsPORB*, OsPORA, *YGL1*, *LYL1* and *CAO* were all down-regulated in *ossac3* when compared with the WT (Figure 5B) [24]. Moreover, *OsR498G0613340000.01* encoding phytoene synthase, *OsR498G0204307900.01* encoding phytoene desaturase (PDS), *OsR498G0101403500.01* encoding lycopene epsilon cyclase (LCYE) and *OsR498G1019092700.01* encoding carotene hydroxylase (CHY) were all involved in carotenoid metabolism, and all of them showed reduced expression level in the *ossac3* mutant when compared with the WT (Figure 5B) [25]. These results indicated that photosynthetic pigment metabolism was severely damaged which was consistent with the result of photosynthetic pigment content measurement [11]. *PsaG*, *PsaK*, *PsaL*, *PsaO* and *PsaN* of photosystem I (PS I) and *PsbO*, *PsbP*, *PsbQ*, *PsbR*, and *Psb27* of PS II showed decreased expression levels in *ossac3* (Figure 5C) [26,27]. Moreover, both *Lhca1*-*4* encoding light-harvesting complex of PSI and *Lhcb2*-*6* encoding light-harvesting complex of PSII showed reduced expression in *ossac3* (Figure 5C) [26]. Additionally, the down-regulated expression pattern of photosynthesis and photosynthetic pigment metabolism-related genes were confirmed by qRT-PCR in *ossac3* (Supplementary Figure S1). These results indicated that photosynthesis was destroyed in *ossac3*, which was consistent with the photosynthesis measurement [11].

### 2.8. Mutation of OsSAC3 Alters Expression of Sugar Metabolism/Distribution Related Genes

Due to sugar accumulation in *ossac3′s* leaves, the different expression pattern of genes in sugar metabolism/distribution between the *ossac3* and WT was analyzed. Additionally, we found that several genes previously reported to participate in sugar metabolism/distribution are differentially regulated in *ossac3* (Figure 5D). Genes participating in starch synthesis (e.g., ADP-Glucose Pyrophosphorylase/AGPase) or sucrose degradation (e.g., UDPG pyrophosphorylase1/UDPGT, Sucrose phosphate synthase/SPS, Sucrose synthase 4/SUS4, Trehalose 6-phosphate phosphatase/TPP and Trehalase) are well represented in our dataset (Figure 5D) [28,29].

Sugar Will Eventually be Exported Transporters (SWEETs) are major transporters mediating sugar flux across cellular membranes in both prokaryotes and eukaryotes [30]. The rice SWEET family harbours 21 SWEET paralogs and are involved in different physiological processes in multiple tissues of the crop plant development [30,31]. OsSWEET3a (*OsR498G0510017200.01*) and OsSWEET6a (*OsR498G0101499300.01*) are essential for hexose translocation, and OsSWEET14 (*OsR498G1120210200.01*) is important for sucrose translocation in phloem loading and seed filling [32,33]. *OsSWEET3a*, *OsSWEET6a* and *OsSWEET14* exhibit reduced expression in the *ossac3* mutant (Figure 5D).

## 3. Discussion

In this study, *OsSAC3* was map-based cloned and identified, and the redox balance, photosynthesis and sugar metabolism/distribution were affected when *OsSAC3* was mutated. It has been reported that two paralogous genes (*AtXDH1* and *AtXDH2*) encode xanthine dehydrogenase in *Arabidopsis* [10,34]. *AtXDH2* was expressed constitutively, and was proposed to play a more general and constitutive function during purine degradation [10,34]. *AtXDH1* transcripts increased at various stresses such as drought, salinity, cold and natural senescence, which indicated its role in stress response. However, *OsSAC3* is single-copied by searching the rice genome and is highly conserved in the eukaryotes, including vertebrates and chlorophytes. Mammalian xanthine dehydrogenase can be converted to xanthine oxidase by modification of cysteine residues or by proteolysis of the enzyme polypeptide chain and four Cys residues were modified to form two disulfide bonds during this conversion of XDH to XO from bovine, rat liver [12,35]. These four Cys residues were all substituted in AtXDH1/2 which indicated that AtXDH1 could not be converted into the xanthine oxidase. Additionally, AtXDH1 was demonstrated to be a strict dehydrogenase and not an oxidase, but was able to produce superoxide radicals [34]. Similar to AtXDH1, those four Cys residues were also missing in OsSAC3 by multiple-sequence alignment.

Two non-identical [2Fe-2S] clusters (Fe/S I and II) were found in xanthine dehydrogenase and provided an electron transfer pathway from molybdenum to FAD [14,36]. Eight strictly conserved Cys residues in the [2Fe-2S] domain was shown to serve as ligands to the two [2Fe-2S] clusters in the xanthine dehydrogenase [14]. Four N-terminal Cys residues displayed the Fe/S II signal, whereas the unusual C-terminal -Cys-Xaa2-Cys-//-Cys-Xaa1-Cys- motif displayed the Fe/S I signal [14]. His124 was also strictly conserved and located near the first Cys residue in the Fe/S I domain. Thus, the electron transfer pathway might be damaged when His124 was mutated to Leu, and this mutation resulted in function loose of *OsSAC3* which led to reduced uric acid content in *ossac3*.

Uric acid is vital for the growth and development of animals and plants. In humans, both uric acid and urate are accumulated in the form of calculi in the joints and/or connective tissues causing arthritis and rheumatic pain [37]. The uriate oxidase-knockout mice spontaneously developed hyperuricemia with about 40% survival up to 62 weeks. Renal dysfunction and glomerula/tubular lesions were also observed in these uricase-knockout mice [38]. The accumulation of uric acid considerably increases the free radical-scavenging activity and resistance against ultraviolet-induced oxidative stress in laboratory-maintained termites [39]. In *Arabidopsis*, accumulated uric acid caused by the defects of urate oxidase (*uox*) also inhibited seed germination, cotyledon development and nutrient mobilization [40]. Thus, the reduced uric acid content caused by the mutation of *OsSAC3* might be the main reason why the growth and development of *ossac3* was inhibited. In addition, uric acid, an important antioxidant, is known to exhibit strong free-radical-scavenging activity in humans, birds, several insects and plants [39,41]. Reduced uric acid might also be the main reason causing accumulated ROS in *ossac3*.

Redox regulation was reported to regulate the activity of starch degradation enzymes [42,43]. α-glucan, water dikinase (GWD1), catalyzes the phosphorylation of starch through a dikinase-type reaction mechanism. GWD1 enzymes are inactive in their oxidized form [44]. The dual-specificity phosphatase *starch excess* 4 (*SEX4*) was required for glucan dephosphorylation; its enzymatic activity was active when reduced, and inactive when oxidized [45]. β-amylase is an exohydrolase that acts at the nonreducing ends of α-1,4-linked glucan chains to produce β-maltose. BAM1, a plastid-targeted β-amylase of Arabidopsis thaliana is specifically activated by reducing conditions [46]. AMY3, an α-Amylases, which cleave α-1,4-glucosidic bonds in starch, is inactive in its oxidized form and could be reactivated by reduced thioredoxins [47]. Thus, the enzymatic activity of GWD1, SEX4, BAM1 and AMY3 would be inactivated in *ossac3* with accumulated ROS. Starch degradation would be inhibited, and this would be the main reason of increased starch observed in leaves containing *ossac3*. The leaves containing *ossac3* also displayed higher sucrose when compared with the wild type [11]. *OsSWEET3a*, *OsSWEET6a* and *OsSWEET14* belonged to the SWEET family; they are seven-transmembrane-domain uniporters that transport hexoses and sucrose and mediate sugar efflux into the apo-plasma [30]. *OsSWEET3a* is reported to transport glucose in the young leaves, and *OsSWEET14* is known to act as a plasma-membrane-localized sucrosea transporter [30]. Thus, the efflux of sugars (mainly sucrose) from the source leaves would be inhibited owing to the decreased expression of *OsSWEET3a*, *OsSWEET6a* and *OsSWEET14*, which would be the main reason that caused the accumulation of sucrose in the early senescenced leaves in the *ossac3*. The increased expression of the AGPase encoding gene *OsR498G0511403400.01* might be induced by the increase in sucrose in the *ossac3* mutant, since increased sucrose could promote starch synthesis by inducing the rate-limiting starch synthetic enzyme AGPase [48]. The granum lamella might be damaged, which resulted from the large amount of starch accumulation, leading to the early senescence phenotype in the *ossac3* mutant. These results indicated that *OsSAC3* played a role in leaf senescence by regulating carbon metabolism.

Previous studies showed that XDH participated in plant growth and development by via purine metabolism. Heterologous overexpression of *VvXDH* in *Arabidopsis* demonstrated the role of *VvXDH* in conferring salt stress by increasing allantoin accumulation and activating the ABA-signaling pathway, enhancing ROS scavenging in transgenic *Arabidopsis* [49]. The XDH-knockdown mutants show significantly reduced tolerance to drought-shock stress [50] and knocking out *AtXDH1* alone enhances the sensitivity to extended darkness [6]. The increased stress sensitivity of these XDH-impaired *Arabidopsis* might be attributable to the deficiency of certain purine metabolites because the application of exogenous urate or allantoin rescued the XDH-knockdown/knockout phenotype [6,10,50]. Moreover, XDH-suppressed lines were subjected to drought stress, plant growth was markedly reduced in conjunction with significantly enhanced cell death and H_2_O_2_ accumulation. This drought-hypersensitive phenotype was reversed by pretreatment with exogenous uric acid, the catalytic product of XDH [50]. Reduced uric acid content and differentially expressed genes related to purine metabolism in the *ossac3* mutant demonstrated that *OsSAC3* played a vital role in the purine/nitrogen metabolism. In *ossac1*, chloroplast grana lamella in mesophyll cells was destroyed because of increased starch granule; thus, the leaves turned yellow and pre-matured. The phenotype of *ossac3* was different from that of typical premature mutants, e.g., *sgr*, *nyc1*, but was similar to that of *ossac1* [5]. In *ossac3*, increased starch granules were also observed in the chloroplast resulted in destroyed grana lamella; then, the leaves turned yellow and pre-matured. *OsSAC1* encoded an endoplasmic reticulum protein of unknown function with two function unknown domains DUF4220 and DUF594 which might function in carbohydrate distribution by regulating plasmodesmata permeability [5]. However, *OsSAC3* was demonstrated to encode the classic xanthine dehydrogenase in our study. We speculated that *OsSAC3* also played a role in leaf senescence by regulating oxidized inhibition of starch degradation and SWEET-mediated carbohydrate distribution in the leaves.

## 4. Materials and Methods

### 4.1. Plant Materials

In this study, Jinhui 10 plant was used as the wild type (WT). The *ossac3* mutant was identified by Huang et al., 2018. The *ossac3* mutant was crossed with Xinong 1B. The Jinhui10, *ossac3* mutant, F_1_ plants, and F_2_ mapping population were cultivated under natural conditions in an experimental field at the Southwest University Rice Research Institute.

### 4.2. Fine Mapping and Isolation of the OsSAC3 Gene

The F_1_ hybrids between Xinong 1B and *ossac3* were constructed and planted. A total of 450 recessive mapping plants from the F_2_ population were used for fine mapping and isolation of the *OsSAC3* gene. Based on the foundations of Huang et al. (2018), 4 polymorphic SSR markers were developed in the 374.2 kb region between SSR marker RM3400 and RM15281. Gene prediction was acquired by searching the Gramene database (http://www.gramene.org/, accessed on 1 January 2018) and the Rice Genome Annotation Project (http://rice.plantbiology.msu.edu/index.shtml, accessed on 1 January 2018). Protein structure analysis was applied in SMART (http://smart.embl.de/smart/change_mode.pl, accessed on 1 January 2021).

### 4.3. Physical and Chemical Analysis

To measure the uric acid content, the middle portion of the flag leaf of the WT and *ossac3* mutant were collected and ground into powder in the liquid nitrogen. Uric acid content measurement was conducted following the manufacturer’s protocol (DIUA009, BioAssay System, USA). Chlorophyll Fluorescence was measured by DUAL-PAM-100 (Walz, Germany).

### 4.4. Multiple Sequence Alignment and Evolutionary Analysis

Protein sequences applied in multiple sequence alignment and phylogenetic tree construction were acquired by searching the Phytozome (https://phytozome-next.jgi.doe.gov/, accessed on 1 January 2021) and NCBI (https://www.ncbi.nlm.nih.gov/, accessed on 1 January 2021) using the *OsSAC3* amino acid sequence as a query. Multiple sequence alignment was performed with ClustalX software using multiple alignment modes. Additionally, the phylogenetic tree was constructed by using the Maximum Likelihood method and JTT matrix-based model. The tree with the highest log likelihood (−35,556.25) is shown. The initial trees for the heuristic search were obtained automatically by applying Neighbor-Join and BioNJ algorithms to a matrix of pairwise distances estimated using a JTT model, and then selecting the topology with superior log likelihood value. The tree is drawn to scale, with branch length measured in the number of substitutions per site. This analysis involved 31 amino acid sequences. There were 1470 positions in the final dataset. Evolutionary analysis was conducted in MEGA X (11.0.11).

### 4.5. Vector Construction, Transformation and Identification

To conduct the complementation test, the WT *OsSAC3* CDS fragment was cloned into binary pCAMBIA1301 under the control of the *CaMV35S* promoter. The accuracies of the constructs were confirmed by sequencing, and the constructs were transformed into the *ossac3* callus mediated by *A tumefaciens* EHA105 following a previously published method. Transformants were screened out under hygromycin and verified by GUS activity detection and sequence.

To conduct the subcellular location analysis of OsSAC3, the WT *OsSAC3* CDS fragment were cloned into the pAN580-GFP vector between the *Spe* I and *Xbal* I sites. The fusion construct and the pAN580-GFP vector were transferred into rice protoplasts by PEG-mediated transformation, respectively. Fluorescence was detected using a confocal laser scanning microscope LSM800 (Zeiss, Oberkochen, Germany).

### 4.6. Gene Expression Analysis

The gene expression pattern was determined by collecting and analyzing samples of the roots, young leaves, old leaves, leaf sheathes, stems and panicles in both the WT and *ossac3*. Total RNA extracted from each tissue and purified following a previously published method [5]. cDNA was reverse-transcribed from the total RNA using a gDNA Eraser kit (Perfect Real Time) (Takara). qRT-PCR was performed using a SYBR Premix *Ex*Taq II (Tli RNaseH Plus) kit (Takara) with a CFX connect Real-time PCR system (Bio-rad, CA, USA). The gene expression data were normalized using the rice *Actin* (LOC_OS03G50885). Primers used in this study are listed in Supplementary Table S2.

### 4.7. Transcriptome and KEGG Pathway Analysis

Total RNA was isolated from the middle of the flag leaf of the WT and *ossac3* with three biological replicates. Library preparation and sequencing of six libraries were conducted by the Novogene (Beijing, China) using a NovaSeq 6000 platform (Illumina, San Diego, CA, USA), following the manufacturer’s protocol. The raw reads were filtered and then mapped to the rice reference genome using Hisat2 (v2.0.5) software with default parameters. Gene expression levels were quantified using StringTie and expressed as fragments per kilobase of transcript per million mapped reads (FPKM). Differential expression analysis of two conditions/groups (two biological replicates per condition) was performed using the DESeq2 R package (1.20.0). The resulting *p*-values were adjusted using Benjamini and Hochberg’s approach for controlling the false discovery rate. padj < 0.05 and |log2(foldchange)| > 1 were set as the threshold for significantly differential expression. The DEGs were used for KEGG pathway analysis and the clusterProfiler R package (3.8.1) to test the statistical enrichment of differential expression genes in KEGG pathways.

## Data Availability

Not applicable.

## Supplementary Materials

The following supporting information can be downloaded at: https://www.mdpi.com/article/10.3390/ijms231911053/s1.
