# Supplementary material for: Mutation of OsSAC3, Encoding the Xanthine Dehydrogenase, Caused Early Senescence in Rice
Source: Int J Mol Sci. 2022 Sep 21;23(19):11053. doi: 10.3390/ijms231911053 (PMC9569572; doi:10.3390/ijms231911053)
Supplement: Supplementary file 1 [file ijms-23-11053-s001.zip › Supplementary Figure S1 Expression pattern of photosynthesis and photosynthetic pigment metabolism related genes between the wild type (WT) and the ossac3 mutant..pptx]

## Slide 1
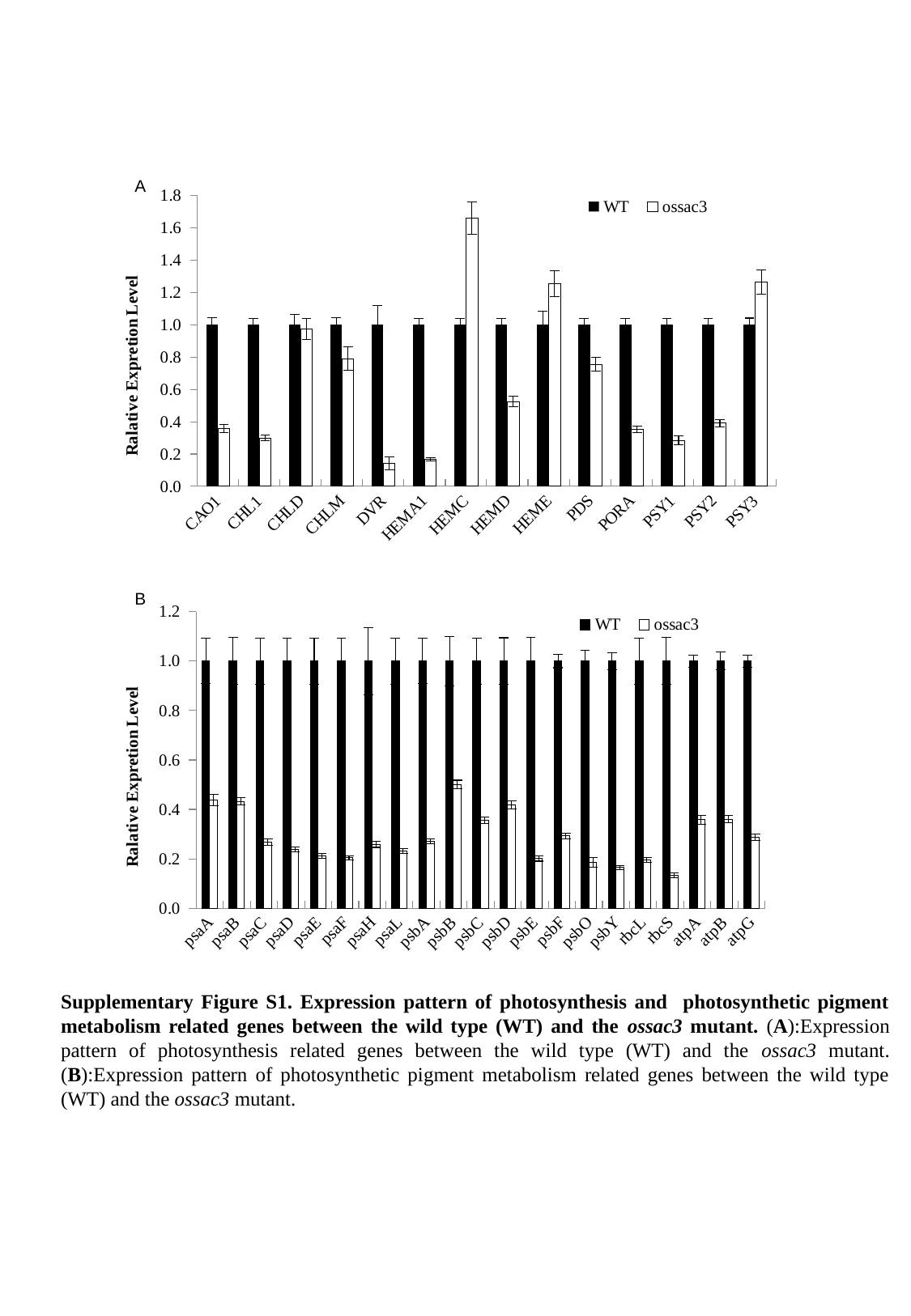

### Chart
| Category | WT | ossac3 |
|---|---|---|
| CAO1 | 1.0 | 0.358609073003839 |
| CHL1 | 1.0 | 0.298805222126998 |
| CHLD | 1.0 | 0.974277948702058 |
| CHLM | 1.0 | 0.790472849568033 |
| DVR | 1.0 | 0.14156131398493 |
| HEMA1 | 1.0 | 0.168216696195921 |
| HEMC | 1.0 | 1.66263730792024 |
| HEMD | 1.0 | 0.525303629140282 |
| HEME | 1.0 | 1.25569910455177 |
| PDS | 1.0 | 0.756153542656439 |
| PORA | 1.0 | 0.353311140238608 |
| PSY1 | 1.0 | 0.284563726480287 |
| PSY2 | 1.0 | 0.390901022687771 |
| PSY3 | 1.0 | 1.2644528877176 |A
### Chart
| Category | WT | ossac3 |
|---|---|---|
| psaA | 1.0 | 0.437697453939429 |
| psaB | 1.0 | 0.431945274591518 |
| psaC | 1.0 | 0.267377754927049 |
| psaD | 1.0 | 0.238780417027769 |
| psaE | 1.0 | 0.212326468183583 |
| psaF | 1.0 | 0.20352133254362 |
| psaH | 1.0 | 0.257990470449044 |
| psaL | 1.0 | 0.231137128027019 |
| psbA | 1.0 | 0.271342782175466 |
| psbB | 1.0 | 0.499905634721362 |
| psbC | 1.0 | 0.35632285585751 |
| psbD | 1.0 | 0.418258094879844 |
| psbE | 1.0 | 0.200990238691896 |
| psbF | 1.0 | 0.292725846183825 |
| psbO | 1.0 | 0.185149720893474 |
| psbY | 1.0 | 0.165133629139018 |
| rbcL | 1.0 | 0.19475287572473 |
| rbcS | 1.0 | 0.134289019422684 |
| atpA | 1.0 | 0.35791942468564 |
| atpB | 1.0 | 0.360297124903918 |
| atpG | 1.0 | 0.286947576309358 |B
Supplementary Figure S1. Expression pattern of photosynthesis and photosynthetic pigment metabolism related genes between the wild type (WT) and the ossac3 mutant. (A):Expression pattern of photosynthesis related genes between the wild type (WT) and the ossac3 mutant. (B):Expression pattern of photosynthetic pigment metabolism related genes between the wild type (WT) and the ossac3 mutant.
